# Design, Synthesis and Biological Evaluation of Medicinal Potential Compounds

**DOI:** 10.3390/molecules31081276

**Published:** 2026-04-13

**Authors:** Renato E. F. Boto, Paulo Almeida, Samuel M. Silvestre

**Affiliations:** RISE-Health, Faculty of Sciences, University of Beira Interior, Rua Marquês de Ávila e Bolama, 6201-001 Covilhã, Portugal; pjsa@ubi.pt (P.A.); sms@ubi.pt (S.M.S.)

In medicinal chemistry, the main objectives are drug discovery and the development of new therapeutic agents. Although great progress has been achieved over the last decades, there are many diseases with no adequate, safe, and effective treatment. Drug resistance has become a critical problem for many drugs already in use. So, the design and synthesis of new lead compounds, as well as their biological evaluation, are still fundamental to the development of drugs acting on biological targets relevant to the diseases [1,2,3,4].

Bioactive molecules can arise from a variety of sources. Although natural products and their derivatives, due to their structural diversity, have been a rich source of bioactive molecules for centuries, modern synthetic chemistry has the capability to prepare rationally designed novel structures, often inspired by nature but optimized for higher efficacy and selectivity as well as improved pharmacokinetic properties. With the vast amount of information being generated in the field of bioactive molecules, computational tools are becoming indispensable for many tasks, such as the design of the molecules, including in silico studies of protein–ligand interactions, and lead optimization, guiding the identification of promising candidates [3,5,6,7].

This Special Issue entitled “Design, Synthesis and Biological Evaluation of Medicinal Potential Compounds” aims to reflect some of the recent developments achieved in this multidisciplinary field. The articles included in this issue illustrate some of the strategies currently employed in medicinal chemistry, ranging from computational and synthetic chemistry approaches to structural characterization of synthesized compounds and biological evaluation of their medicinal potential. This Special Issue includes ten articles dealing with a wide range of biological targets and therapeutic areas, reflecting the importance of design and synthesis on the search for bioactive compounds.

The first article of this issue, by Akhmetova et al., presents the synthesis of a new class of *N*-substituted piperidine derivatives with possible antiviral activity [Contribution 1]. The studied compounds were obtained by a cyanohydrin synthetic approach aiming at the preparation of some piperidinecarboxylic acids and their 1D and 2D NMR characterization. The in vitro antiviral activity of the compounds studied was examined by using the influenza A/Swine/Iowa/30 (H1N1) virus and revealed promising antiviral activity, suggesting that this scaffold may provide useful leads for further optimization.

For the development of a synthetic resveratrol-type analog [Contribution 2], designated as RE-1, Lin et al. employed natural product-inspired drug design. The objective was to target the VEGF signaling pathway, which is commonly associated with a variety of diseases, including cancer. This compound showed an enhanced inhibition of VEGF-associated activity compared to that of its parent compound, resveratrol, in several biological assays, emphasizing the potential of rationally modified natural scaffolds.

Huang et al.’s [Contribution 3] study evaluates the effects of resveratrol butyrate esters and derivatives on the oxidative metabolism of trimethylamine (TMA) and the expression of flavin-containing monooxygenase-3 (FMO3) gene in cultured human cells and bacteria. This study identifies several compounds that regulate TMA metabolism and highlights the potential of dietary supplementation with resveratrol butyrate esters and derivatives as a means of reducing the levels of trimethylamine oxide (TMAO), a metabolite that has been linked to cardiovascular disease.

Yuan et al. [Contribution 4] presented a library of 2-arylpropanoic acid-L-tryptophan derivatives as potential protective agents against cisplatin-induced nephrotoxicity. One of the derivatives had the most desired effects, namely, increased cell viability of renal tubular epithelial cells, and was also found to inhibit the expression of several inflammatory biomarkers that are associated with kidney injury. The effects of this compound on these biomarkers were confirmed by molecular docking, which suggests that the compound has anti-inflammatory activity by targeting the gastrin-releasing peptide receptor (GRPR), an important regulator of inflammation, highlighting the therapeutic relevance of this compound.

Lv et al. [Contribution 5] reported the design, synthesis, and biological evaluation of a series of aspartic acid derivatives as potential antifibrotic drugs. These derivatives were synthesized from L-aspartic acid, and the lead candidate showed remarkable ability to suppress the COL1A1 promoter and decrease fibrosis-related genes and proteins in liver cell models, bringing promise for the treatment of liver fibrosis.

This Special Issue also includes peptide-based approaches, illustrated in the article by Angelova et al. [Contribution 6]. This article describes the synthesis of new analogs of the broad-spectrum antimicrobial peptide aurein 1.2 containing non-proteinogenic amino acids in order to increase its bioactivity. The new analogs showed an improved bioactivity compared to the native aurein 1.2 peptide, and one of them displayed exceptional antimicrobial activity against all assayed bacteria as well as on two cancer cell lines.

Although these studies investigate different biological targets and/or bioactivities, they all follow the same principle: taking the structural knowledge of successful molecules and optimizing them to obtain better biological activity. This can be achieved by optimizing small-molecule leads or by re-engineering peptide structures, which in both cases can translate into improved biological activity.

Maia et al. [Contribution 7] have explored the antiproliferative effects of some asymmetric cyanine dyes on Caco-2 human colorectal adenocarcinoma cells. Several new synthesized dyes showed promising efficacy and selectivity towards colorectal cancer cells in in vitro assays. The possible involvement of the topoisomerase II enzyme, in addition to the apoptosis induction, has been suggested.

Research focused on a neglected human tropical disease was conducted by Vásquez et al. [Contribution 8]. They synthesized and evaluated quinazoline nitro-derivatives as lead compounds for the development of new drugs for the treatment of Chagas disease, a tropical disease caused by the parasite *Trypanosoma cruzi*. In fact, several nitro-derivatives showed significant in vitro activity against the parasite and low toxicity to human foreskin fibroblast cells.

This Special Issue also highlights the work of Islam et al. [Contribution 9] on a class of thiazolo [4,5-*d*]pyrimidine derivatives that behave as selective antagonists of the corticotropin-releasing factor receptor 1 (CRF_1_R). Some analogs have higher affinity for this receptor than the known antagonist antalarmin and therefore emphasize the potential of this class of molecules for treating stress-related disorders and congenital adrenal hyperplasia.

Finally, Shulgau et al. [Contribution 10] synthesized thiourea derivatives based on 3-aminopyridin-2(1*H*)-one scaffolds and evaluated their activity as α-glucosidase inhibitors in the context of diabetes mellitus. Some of the synthesized derivatives exhibited better inhibitory activities than the reference drug acarbose and, hence, possess good potential for designing novel antidiabetic agents.

Taken together, the contributions presented in this Special Issue illustrate some recent advances in contemporary medicinal chemistry research. The diversity of research areas and biological targets/diseases in each individual article may appear large, but there is a common thread running through all of them: the design and synthesis of molecules with a defined profile, the assessment of their biological activity, and the exploration of their medicinal potential with the aim of developing drug candidates that are more effective and safer than current therapies. Such multidisciplinary efforts are fundamental to increasing the number of promising drug candidates available to researchers and to accelerating the process of clinical development, and thus to bringing new treatments.

The articles in this Special Issue illustrate clearly that progress in the discovery of new scientific knowledge hardly ever occurs in a straightforward, planned, and linear fashion. In fact, adequate design and planning are very important for medicinal chemistry. However, the actual advances in this field are almost always the result of a process of testing, refining, and revising hypotheses, during which failures are often seen as hurdles, but are in fact essential to the advancement of the field. It also illustrates very effectively how medicinal chemists of all levels of expertise, from experienced senior scientists to postdoctoral researchers and graduate students, can work together towards achieving a common goal.

In this context, the words of the Portuguese poet Fernando Pessoa seem particularly fitting: “*Tudo vale a pena quando a alma não é pequena*” (“*Everything is worthwhile when the soul is not small*”), from *Mensagem* [8]. This sentiment summarizes the essence of scientific endeavor, emphasizing that sustained curiosity, perseverance, and intellectual generosity can transform uncertain or challenging paths into meaningful progress.
